# Reduction Pathway and Temperature-Dependent Decomposition of Epitaxial BiFeO_3_ Thin Films Under CaH_2_ Treatment

**DOI:** 10.3390/ma19071310

**Published:** 2026-03-26

**Authors:** Jie Gong, Nian Li, Mahliya Lokman, Mengsha Li, Ke Zhang, Liang Qiao

**Affiliations:** 1 School of Physics, University of Electronic Science and Technology of China, Chengdu 611731, China; jiegong@std.uestc.edu.cn (J.G.); liang.qiao@uestc.edu.cn (L.Q.); 2 Center for Microscopy and Analysis, Nanjing University of Aeronautics and Astronautics, Nanjing 210016, China; leah05@nuaa.edu.cn

**Keywords:** CaH_2_ reduction, BiFeO_3_ thin films, oxygen vacancies, chemical decomposition, valence reconstruction

## Abstract

The control of oxygen stoichiometry via topochemical reduction offers a powerful route to manipulate the functional properties of complex oxides. Here, we investigate the chemical and structural evolution of epitaxial BiFeO_3_ (BFO) thin films under CaH_2_ treatment in a sealed tube, using a representative reduction condition of 365 °C for 2 h and a temperature window of 345 to 380 °C to probe the reduction dependent evolution. The inherent sensitivity of BFO’s multiferroic properties to oxygen vacancy formation and cation valence states makes it an ideal platform to probe reduction pathways. The aim of this work is to elucidate the detailed reduction pathway, including phase stability, valence changes in Bi and Fe, and the morphological consequences of oxygen extraction. Using a combination of spectroscopic, diffraction, and microscopic techniques, it was demonstrated that CaH_2_ annealing does not yield a homogeneous oxygen-deficient perovskite. Instead, it triggers a decomposition into Bi_2_O_3_, metallic Bi, and FeO*_x_* secondary phases, accompanied by severe surface roughening. This chemical reconstruction leads to a strong suppression of the ferromagnetic-like response and a redshift in the optical absorption edge.

## 1. Introduction

Transition-metal perovskite oxides (ABO_3_) have attracted tremendous attention in modern solid-state chemistry and materials physics, owing to remarkable spectrum of electronic, magnetic, and ferroic functionalities. The deliberate tuning of these properties has long been pursued through chemical substitution at the cation sites (A or B), effectively altering the local charge balance and electronic bandwidth [1,2]. A classic paradigm is La_1-*x*_Sr*_x_*MnO_3_, where the Mn^3+^/Mn^4+^ ratio is precisely controlled via aliovalent doping, governing its colossal magnetoresistance [3,4].

In contrast, the direct and continuous modulation of the anion sublattice—particularly oxygen stoichiometry—offers a complementary and potent chemical strategy for property engineering. Oxygen vacancies ($V_{O}^{\cdot \cdot }$) are not merely point defects; they act as potent dopants, altering carrier concentration, lattice strain, and the crystal field environment of transition-metal ions, thereby modifying orbital occupancy and spin interactions [5,6]. However, conventional methods for introducing $V_{O}^{\cdot \cdot }$, such as high-temperature annealing in reducing atmospheres or vacuum, are often thermodynamically brutal [7,8]. They typically lead to broad phase distributions, uncontrolled decomposition of metastable phases, or irreversible damage to delicate epitaxial structures, providing limited, non-selective tunability [9].

Topochemical reduction has emerged as a refined “soft chemistry” tool. This approach employs low-temperature (~300–400 °C) reactions with solid-state metal hydrides (e.g., NaH, CaH_2_) as reducing agents [8]. The process can be viewed as an intercalation of H^−^ (or its equivalent) followed by the de-intercalation of O^2−^, often expressed as: ABO_3_ + *x*H^−^ → ABO_3-*x*_ + *x*OH^−^ (→H_2_O↑). Its key advantage lies in its potential for topotactic transformations, where the fundamental structural framework of the parent oxide is largely preserved, while the oxygen content and associated transition-metal valence states are selectively and homogeneously tuned [10,11]. This method has unlocked new horizons in oxide chemistry, enabling the synthesis of metastable phases inaccessible by conventional means. Seminal examples include the reduction of perovskite nickelates to infinite-layer phases, which unveiled new superconducting states [12], and the creation of anion-ordered brownmillerites from simple perovskites, showcasing precise oxygen vacancy engineering [13,14]. These achievements highlight topochemical reduction not merely as a synthetic trick but as a fundamental pathway to new electronic ground states.

For oxide thin films, CaH_2_-based topochemical reduction is particularly attractive because it allows oxygen stoichiometry to be tuned under comparatively mild conditions, well below the temperatures typically required in conventional reducing treatments. This feature is especially important for epitaxial films, where crystallographic coherence, interface quality, and surface morphology are often closely linked to functional performance. In principle, such a soft chemical route offers the possibility of accessing oxygen-deficient states while minimizing the extensive structural damage, interdiffusion, and morphological degradation that often accompany harsher reduction processes. At the same time, whether this strategy can be extended in a controlled way to chemically fragile multication oxides remains an open question. This issue is especially relevant for Bi containing perovskite thin films, in which oxygen removal may be coupled to A site instability and chemical reconstruction rather than a simple topotactic reduction pathway.

Hydride-based topochemical reduction is most successful in perovskites with redox-inert A-site cations (typically rare-earth or alkaline-earth ions), where oxygen deintercalation is primarily accommodated by reduction of the transition-metal B-site and a topotactic coordination change, enabling brownmillerite/infinite-layer transformations while preserving the A-site sublattice. Representative examples include LaNiO_3_ → LaNiO_2_, NdNiO_3_ → NdNiO_2_, and SrFeO_3_ → SrFeO_2_ [12,15]. In contrast, BiFeO_3_ contains Bi^3+^ on the A-site, which is comparatively susceptible to reduction and chemical reconstruction under strong hydride conditions; oxygen removal can couple to Bi–O bond destabilization and Bi exsolution, thereby undermining the perovskite scaffold and promoting phase separation rather than a controlled oxygen-deficient perovskite [16]. The literature on Bi-containing oxides and related thin films further suggests that, once the oxygen chemical potential is driven sufficiently low, A-site (Bi) instability, volatility/segregation, and Bi-rich secondary phases can become kinetically accessible, especially near free surfaces and defect-rich regions. These comparisons suggest a practical design rule: hydride topochemical reduction favors perovskites with redox-inert, non-volatile A-site cations and frameworks that tolerate large anion removal, whereas perovskites containing reducible p-block A-site cations (e.g., Bi^3+^) tend to have a narrower stability window and a higher propensity for decomposition once deep reduction is triggered [17]. In this sense, the relative fragility of Bi–O bonding implies a lower μO stability limit under hydride reduction, motivating the use of kinetic/chemical ‘buffers’ (e.g., capping/barrier concepts or milder oxygen getters) when attempting to access oxygen-deficient states without collapse.

Despite these successes, a critical and less explored chemical question persists: What are the stability limits and specific reduction pathways for complex perovskites containing multiple, easily reducible cations? BiFeO_3_ (BFO), a room-temperature multiferroic perovskite, serves as a compelling candidate, due to its intricate and sensitive chemical nature. Its functional properties arise from the cooperative effects of the stereochemically active 6s^2^ lone pair on Bi^3+^ (driving ferroelectricity) and the partially filled d-orbitals of high-spin Fe^3+^ (governing magnetism) [18,19,20]. Consequently, BFO thin films are actively explored for applications in next-generation electronic devices, such as non-volatile memories, magnetoelectric sensors, and photovoltaic components, where defect engineering, including controlled oxygen vacancy formation, is a crucial strategy for performance optimization [21,22,23]. Crucially, both sublattices are chemically vulnerable under reducing conditions: the Fe^3+^/Fe^2+^ redox couple is active, and the Bi–O bond is notably weak, with Bi^3+^ prone to reduction to volatile Bi^0^ [24,25]. This intrinsic chemical duality makes BFO an ideal platform to study whether a multi-cation oxide can sustain a controlled, homogeneous topochemical reduction, or if the process inevitably triggers a cascade of irreversible decomposition reactions driven by the differing redox energetics of its constituent ions.

In this work, CaH_2_-mediated topochemical reduction is employed to interrogate the chemical fate of epitaxial BFO thin films. Using a suite of spectroscopic, diffraction, and microscopic techniques, the study goes beyond cataloging property changes to elucidate the stepwise chemical pathway, phase evolution, and ultimate stability limit of the BFO perovskite under mild reducing conditions. Rather than forming a homogeneous, oxygen-deficient BFO_3−x_ phase, the system undergoes a thermodynamically driven chemical decomposition into binary and elemental secondary phases. This study provides a detailed chemical map of reduction-induced transformation in a prototypical multiferroic oxide, offering crucial insights for the future design of anion-engineered functional materials via soft chemistry routes.

## 2. Experimental Section

Film synthesis. High-quality BFO thin films were grown on SrTiO_3_ (001) substrates by pulsed laser deposition (PLD). Optimal growth conditions were determined through systematic optimization of energy density, oxygen pressure, substrate temperature, annealing temperature, and annealing time. High-quality films were obtained at an energy density of 1.6 J/cm^2^, a substrate temperature of 650 °C, and an oxygen pressure of 0.27 mbar. After deposition, a 3-unit-cell (u.c.) SrTiO_3_ capping layer was deposited under the same oxygen ambience to minimize surface degradation during subsequent handling and reduction treatment. CaH_2_ reduction was performed in a tube furnace (Beiyike 1200C, Anhui Beiyike Equipment Technology Co., Ltd., Hefei, China). The films were wrapped with high-purity Al foil and placed in a flame-sealed quartz tube together with ~0.1 g CaH_2_. The tube was evacuated to ~0.1 mTorr for 10 min before sealing. The sealed tube was heated/cooled at 10 °C min^−1^ to the target temperature (typically 365 °C) and held for 2 h, followed by cooling to room temperature. After opening, the samples were unwrapped and gently blown with high-purity N_2_ prior to immediate characterization to minimize re-oxidation.

Relevant Performance Characterization. Structural characterization was conducted to analyze the crystal structures of both as-grown and reduced films. High-resolution X-ray diffraction (XRD) and reciprocal space mapping (RSM) were performed using a D8 Discover diffractometer equipped with a LYNXEYE-2 line detector (Bruker, Billerica, MA, USA). The system utilized monochromatic Cu-Kα X-rays, produced with a four-bounce Ge (220) double-crystal monochromator combined with a Cu X-ray mirror, providing a beam divergence of 12 arcseconds. A 0.09° parallel plate collimator was used to filter the diffracted beam. Film thickness was determined through X-ray reflectivity (XRR) measurements, with the results modeled using an air/film/substrate interface simulation. The surface morphology and step-terrace structures of the thin films were analyzed using an atomic force microscopy (Park NX10, Park Systems, Suwon, Republic of Korea). X-ray photoelectron spectroscopy (XPS) measurements were carried out using a SPECS system (SPECS Surface Nano Analysis GmbH, Berlin, Germany). All XPS spectra were charge-corrected using the C 1s peak at 284.8 eV. Peak fitting was performed using CasaXPS, version 2.3.27, with a Shirley background and mixed Gaussian-Lorentzian line shapes with constrained peak widths. X-ray absorption spectroscopy (XAS): Fe L-edge and O K-edge XAS spectra were collected in total electron yield (TEY) mode. Raw spectra were background-subtracted and subsequently normalized to a 0–1 intensity scale to facilitate comparison among samples. The magnetic properties, specifically the temperature-dependent magnetization M(T) and the field-dependent magnetization M(H) (hysteresis loops), were measured using a Physical Property Measurement System were measured using a Physical Property Measurement System (PPMS-14T DynaCool, Quantum Design, San Diego, CA, USA). The magnetic signal from a bare SrTiO_3_ substrate (and sample holder background) was measured under the same conditions and subtracted; the remaining M(H) curves were corrected by removing the high-field linear background. To ensure reproducibility, ~200 films were prepared during growth optimization; ~20 films with consistent epitaxial quality were selected for systematic characterization. AFM was measured at five locations per film, and representative magnetometry measurements were repeated three times, yielding consistent trends.

## 3. Results and Discussion

Substrate quality significantly impacts the structure and quality of epitaxial thin films. A smooth, flat substrate surface is crucial for the growth of high-quality films. Therefore, substrate pre-treatment and selection are essential before epitaxial growth. In this work, SrTiO_3_ (001) substrates were etched using buffered hydrofluoric acid (BHF). The etching mechanism is based on the preferential reaction of SrO with BHF compared to TiO_2_, forming water-soluble strontium hydroxide, Sr(OH)_2_. After etching and nitrogen blow-drying, the substrates were annealed in a tube furnace to obtain a step-like surface morphology [26]. Annealing was performed at 1000 °C for 1 h to prevent further Sr diffusion from the bulk to the surface at higher temperatures [27]. This combined wet etching and thermal annealing process induced surface atomic reconstruction, resulting in a clear step-and-terrace structure, as shown in the AFM image of Figure S1a, indicating the formation of a TiO_2_-terminated surface [28]. The step height, determined from Figure S1b, is 0.33 nm. The experience reported in the literature indicates that SrTiO_3_ substrates with a step-and-terrace surface morphology favor the formation of perovskite-phase thin films, whereas substrates with a mixed surface morphology resulting from mechanical polishing readily lead to the growth of secondary phases. Consequently, all substrates used for pulsed laser deposition (PLD) in this study were treated with etching and annealing to achieve a step-and-terrace surface.

**Figure 1:**
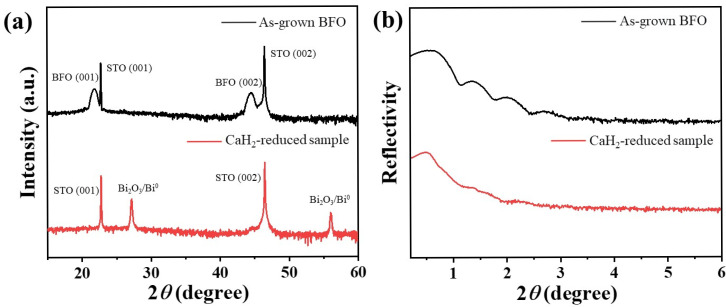
Characterization of epitaxially grown BFO thin films before and after reduction using (**a**) XRD and (**b**) XRR.

The microstructure of BFO films, before and after reduction using calcium hydride, was characterized by thin-film X-ray diffraction (XRD). Figure 1a shows the XRD pattern of the BFO film. The (001) and (002) diffraction peaks of the SrTiO_3_ (001) substrate, along with the (001) and (002) peaks of the BFO film at 21.6° and 44.3°, respectively, are clearly visible. The alignment of these peaks indicates a single-crystal BFO film epitaxially grown on the SrTiO_3_ (001) substrate, with no secondary phases detected [29,30]. The high intensity, sharp shape, and narrow full width at half-maximum (FWHM) of the BFO peaks suggest high-quality crystallinity and a good epitaxial interface with the substrate [31]. After reduction with calcium hydride at 365 °C for 2 h, the sample’s diffraction peaks correspond to bismuth oxide and elemental bismuth, located at 27.5° and 56.0° respectively. As a control, an identical sealed-tube anneal without CaH_2_ shows no detectable secondary-phase reflections and no obvious peak-position changes (Figure S2), indicating that the thermal history alone does not account for the decomposition signatures. This indicates that the crystal structure of the reduced BFO thin film is severely damaged, resulting in a change in its crystal structure and composition. The peak at 27.5° shows overlap because both Bi_2_O_3_ and elemental bismuth exhibit diffraction angles near this 2θ value [32,33]. The additional reflections observed after CaH_2_ treatment are consistent with Bi-containing decomposition products (Bi_2_O_3_ and/or Bi^0^). Because several Bi_2_O_3_ and Bi^0^ reflections are closely spaced in 2θ and the number of film-related peaks is limited in standard θ–2θ thin-film XRD, the present lab XRD data do not uniquely quantify their relative fractions. Therefore, further analysis is necessary to definitively identify this diffraction peak. Figure 1b shows the X-ray reflectivity (XRR) results of epitaxially grown BFO thin films before and after reduction. The XRR pattern of the as-grown BFO film exhibits clear oscillatory fringes (Kiessig fringes), indicating good crystallinity [34]. The film thickness, determined from these oscillations, is 16 nm. Accordingly, the average deposition rate is estimated to be 125 pulses/nm, based on the measured thickness and the total number of laser pulses (2000). After reduction, the oscillatory fringes in the XRR pattern become less distinct. This suggests that the reduction process altered the density of the BFO film and increased surface roughness, leading to reduced reflectivity. Another possibility is that the reduction altered the film’s chemical composition, which would also affect the electron density and thus the reflectivity. These conclusions are consistent with the XRD results.

To further probe the kinetic role of reduction temperature, CaH_2_ treatments were conducted for a fixed duration of 2 h over a temperature range of 345 to 380 °C and collected the corresponding XRD series (Figure S3). With increasing temperature, the perovskite-related diffraction features progressively deteriorate while additional reflections emerge and strengthen, indicating a temperature-accelerated, threshold-like transition rather than a purely binary “before/after” change. This temperature series supports that, under the investigated sealed-tube CaH_2_ treatment conditions, decomposition and phase separation become dominant beyond a critical reduction severity. This behavior indicates a threshold type transition rather than a continuous evolution toward a homogeneous oxygen-deficient phase.

To rationalize the experimentally observed structural degradation from a thermodynamic perspective, first-principles calculations were performed for stoichiometric BiFeO_3_ and an ideal deep oxygen-deficient limit, BiFeO_2_, including analyses of the band structure, density of states, polarization response, and formation enthalpy (Figure S4). Within computational framework, BiFeO_3_ exhibits a clearly favorable (negative) formation enthalpy, whereas the deep oxygen-deficient BiFeO_2_ limit shows a positive formation enthalpy, indicating that deep oxygen removal into a BiFeO_2_-like perovskite is thermodynamically unfavorable and prone to destabilization. This energetic trend supports experimental observations that sufficiently strong CaH_2_ reduction drives collapse and phase separation, highlighting the need to control temperature and time to avoid entering a deep oxygen-deficient regime. BiFeO_2_ is used here as an idealized limiting model to evaluate stability trends, and the actual reduced state may evolve via multiphase pathways.

**Figure 2:**
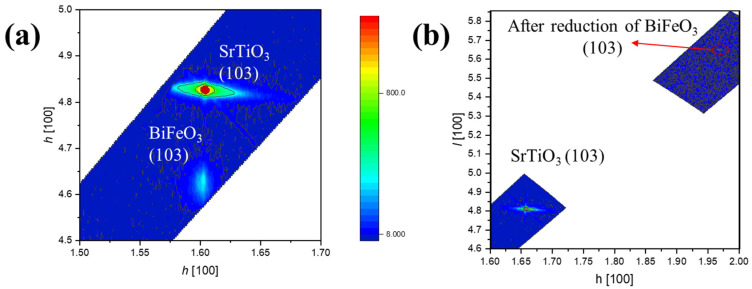
Reciprocal space maps (RSMs) of the (103) plane of BFO thin films (**a**) before and (**b**) after reduction treatment.

To further investigate the epitaxial relationship between BFO thin films and SrTiO_3_ substrates before and after reduction, reciprocal space mapping (RSM) was used to analyze the growth modes of BFO thin films pre- and post-reduction. All reflections are indexed using the pseudocubic (pc) notation, which is a standard convention for epitaxial perovskite thin films. The SrTiO_3_ substrate is indexed in the cubic perovskite setting $(Pm\bar{3}m).$ For the epitaxial BiFeO_3_ film, the (00l)_pc_ reflections correspond to a strain-distorted perovskite unit cell indexed in pseudocubic notation and do not imply that the film is strictly cubic. Figure 2 presents the RSM data obtained by scanning the SrTiO_3_ (103) crystal plane. In Figure 2a, both the SrTiO_3_ substrate and BFO thin film display single diffraction peaks in the mapping, indicating high-quality epitaxial growth of the thin film [35]. The in-plane reciprocal space points of the BFO thin film coincide with those of the SrTiO_3_ substrate, demonstrating that the BFO thin film adopts a fully strained growth mode, with an in-plane lattice constant closely matching that of SrTiO_3_ (a ≈ 3.905 Å). The RSM after reduction is shown in Figure 2b. The intense reduction reaction results in the additional reflections indicative of secondary phases. These secondary phases appear as new peaks in the reciprocal space map, distinct from the original BFO peaks, as highlighted in the red circled regions in Figure 2b. Additionally, lattice distortions caused by the reduction process lead to shifts in the reciprocal space peaks, resulting in corresponding displacements of the BFO (103) diffraction spot.

Atomic force microscopy (AFM) was used to examine the surface morphology evolution of BFO thin films before and after CaH_2_ treatment (Figure S5) [36]. As shown in Figure S5a, the pristine film exhibits a granular surface morphology. The root-mean-square roughness (Rq) extracted from the AFM image is 1.817 nm, indicating nanoscale height variations on the film surface. After reduction, as shown in Figure S5b, the surface morphology changes markedly from an irregular granular texture to a striped pattern. Concomitantly, Rq increases to 2.571 nm, suggesting an appreciable roughening of the surface. This morphological reconstruction is likely associated with reduction-driven changes in oxygen stoichiometry and local bonding, and may also be influenced by near-surface chemical/structural inhomogeneity and the possible precipitation of secondary-phase particles. AFM reveals pronounced surface roughening and non-uniform mesoscale topography after CaH_2_ treatment. AFM alone does not provide PSD/correlation length/periodicity metrics or uniquely determine the origin of stripe-like features; therefore, these observations are discussed qualitatively and interpreted together with XRR and STEM as evidence of surface degradation and chemical reconstruction.

**Figure 3:**
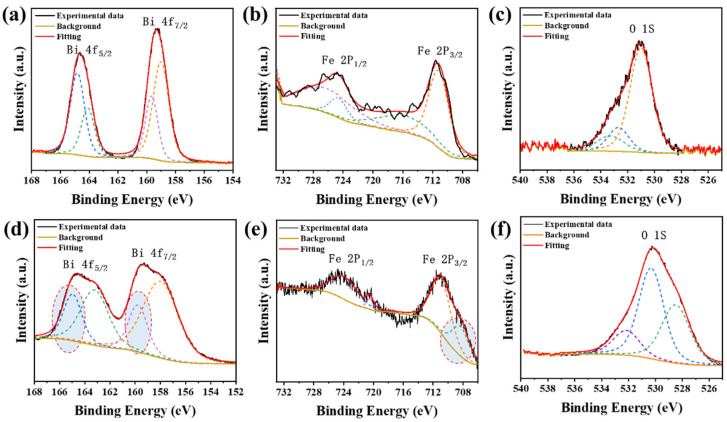
XPS spectra of BFO thin films before (**a**–**c**) and after (**d**–**f**) reduction. The colored dashed lines denote the fitted component peaks, the solid line denotes the overall fit.

As a surface-sensitive technique, XPS is used here to identify qualitative changes in chemical states; quantitative stoichiometry or phase fractions are not extracted from peak areas for the reduced, rough/chemically heterogeneous films. Figure 3 presents the X-ray photoelectron spectroscopy (XPS) core-level spectra of Bi 4f, Fe 2p, and O 1s for BFO thin films before and after CaH_2_ reduction. Before reduction, as shown in Figure 3a, the Bi 4f spectrum exhibits two distinct main peaks, Bi 4f_7/2_ and Bi 4f_5/2_, located at approximately 158.8 eV and 164.1 eV, respectively, corresponding to the Bi^3+^ oxidation state. This indicates that Bi predominantly exists in the Bi^3+^ oxidation state in the thin films, consistent with the chemical composition of BFO [37]. After reduction, as shown in Figure 3d, the intensity of the Bi 4f main peaks significantly decreases, while new peaks emerge at lower binding energies (~156.8 eV and ~162.2 eV), indicating the partial reduction of Bi^3+^ to metallic Bi(0). This transformation aligns with the decomposition of Bi from BFO during the reduction process, resulting in the formation of secondary-phase metallic Bi. The Fe 2p spectrum of the BFO thin films before reduction, as shown in Figure 3b, features an Fe 2p_3/2_ main peak at approximately 710.6 eV and a shake-up satellite peak at 718.7 eV, indicating that Fe is predominantly in the Fe^3+^ oxidation state, consistent with the perovskite structure of BFO [38]. After reduction, as shown in Figure 3e, the Fe 2p_3/2_ main peak shifts to a lower binding energy of 709.8 eV, suggests partial reduction of Fe^3+^ to Fe^2+^ and the possible emergence of Fe^2+^-enriched FeOₓ (FeO-like) species. This binding energy shift reflects the change in the chemical environment of Fe, likely due to the loss of oxygen and the introduction of oxygen vacancies during the reduction process. The O 1s spectrum of the BFO thin films before reduction, as shown in Figure 3c, has a main peak at 529.9 eV, corresponding to lattice oxygen (Bi-O and Fe-O bonds), and a shoulder peak at 531.5 eV, attributed to a small amount of adsorbed oxygen [39]. After reduction, as shown in Figure 3f, the intensity of the O 1s main peak decreases significantly, and the peak slightly shifts to higher binding energies, consistent with oxygen loss and chemical reconstruction. The O 1s envelope can include contributions from lattice oxygen, adsorbates, and oxygen in secondary phases; therefore, the present spectra are discussed qualitatively rather than used to quantify an absolute oxygen deficiency (δ). Additionally, the enhanced shoulder peak at higher binding energies may be associated with adsorbed oxygen species or secondary-phase oxides, such as Bi_2_O_3_. In summary, the XPS analysis clearly reveals the impact of the reduction process on the chemical environment and electronic structure of BFO thin films.

**Figure 4:**
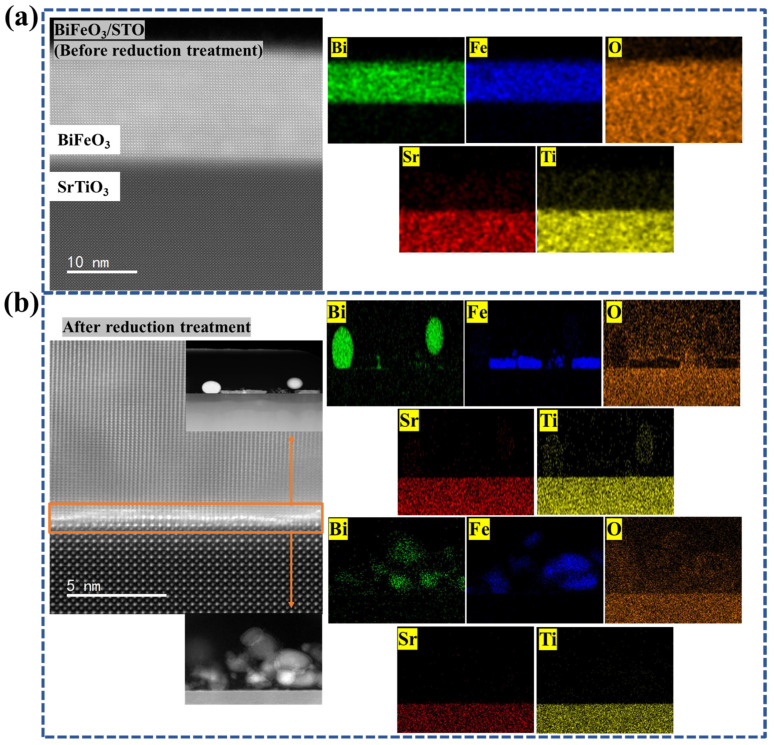
STEM images and EDS elemental maps of BFO thin films: (**a**) before reduction and (**b**) after reduction.

To probe the microstructural evolution and elemental distribution of BFO thin films before and after CaH_2_ treatment, cross-sectional STEM imaging combined with EDS mapping was performed (Figure 4). As shown in Figure 4a, the STEM image of the as grown BFO thin film reveals a clear interface, with the film thickness measured to be approximately 16 nm (around 38 u.c. layers), consistent with the XRR results. No obvious extended defects are observed in the imaged area [40]. The corresponding EDS maps indicate a relatively uniform distribution of Bi, Fe, and O across the film, while Sr and Ti signals remain confined to the substrate, suggesting no obvious interdiffusion at the interface within the detection limit. Note that an ultrathin SrTiO_3_ capping layer was deposited after BFO growth; however, due to the ultrathin nature of this layer and the limited contrast in the topmost region in cross-sectional STEM (further complicated by surface roughening/reconstruction after CaH_2_ treatment and routine specimen preparation), the capping layer is not distinctly resolved in the STEM overview image.

After CaH_2_ treatment (Figure 4b), the film surface becomes markedly roughened, and irregular spherical/flocculent protrusions are observed near the top surface, consistent with the roughness increase revealed by AFM. The EDS maps show pronounced chemical inhomogeneity in the near-surface region: Bi- and Fe-related signals become spatially concentrated in these protrusions, whereas the relative O signal is reduced. Given the limited quantitative sensitivity of EDS to light elements and the influence of local topography, these maps are interpreted qualitatively as evidence of oxygen loss accompanied by cation segregation/precipitation during reduction. In contrast, the region close to the SrTiO_3_ interface remains comparatively continuous with more uniform elemental distribution, suggesting that part of the original BFO layer is preserved near the substrate. Notably, the cross-sectional STEM–EDS results suggest a depth-dependent reduction behavior. The near-surface region exhibits pronounced morphological roughening and chemical inhomogeneity, whereas the interface-proximal region remains comparatively continuous with a more uniform elemental distribution, indicating that the reduction is more severe toward the surface and less pronounced near the substrate. Given the limited quantitative sensitivity of EDS to light elements (especially oxygen), this interpretation is qualitative; therefore, these observations are described as consistent with a surface-initiated, kinetics-limited reduction process and an interface-stabilized residual layer, rather than a fully uniform reduction throughout the thickness. Taken together with XRD and XPS, the STEM–EDS results support reduction-driven decomposition and secondary-phase formation predominantly in the near-surface region, which disrupts the initial smooth epitaxial architecture.

While oxygen loss is central to the reduction process, a reliable quantitative δ cannot be extracted from the present dataset because XPS probes only the near-surface region and the reduced films are rough/chemically heterogeneous, Fe L-edge XAS does not uniquely determine oxygen stoichiometry, and STEM–EDS is not quantitatively robust for oxygen due to limited sensitivity and topography/thickness effects.

STEM–EDS further indicates severe oxygen loss predominantly in the near-surface region. This creates a strong vertical chemical gradient, with the interface proximal region retaining a more uniform BFO-like composition. This gradient suggests a surface-initiated reduction mechanism, where kinetics are limited by diffusion through the evolving decomposition layer, and the epitaxial interface may partially stabilize the perovskite structure against complete breakdown.

**Figure 5:**
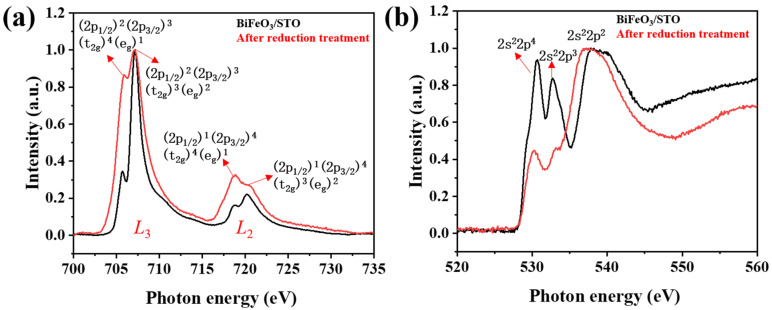
X-ray absorption spectroscopy (XAS) of BFO thin films before and after reduction. The black curves correspond to the pristine film, and the red curves correspond to the reduced film: (**a**) Fe L-edge; (**b**) O K-edge.

To investigate the changes in the electronic structure and chemical states of Fe and O elements in BFO thin films before and after reduction, X-ray absorption spectroscopy (XAS) was used to perform a detailed analysis of the local electronic environment. As shown in Figure 5, the XAS data of Fe and O elements in BFO thin films before (black curve) and after (red curve) reduction reveal distinct variations. As depicted in Figure 5a, the Fe L-edge spectrum of the BFO thin film before reduction exhibits characteristic Fe^3+^ peaks corresponding to Fe 2p → 3d transitions. The L_3_ edge (approximately 710 eV) and L_2_ edge (approximately 723 eV) display two strong peaks, indicating that Fe primarily exists in a high-valence state (Fe^3+^), consistent with the chemical environment of Fe in the perovskite structure of BFO. Additionally, the spectrum’s high symmetry and resolution reflect a uniform chemical environment for Fe in the thin film [41]. After reduction, the main peak positions of the L_3_ and L_2_ edges shift slightly toward lower energy (~0.5 eV), and the spectral line shape evolves, showing reduced peak intensity and the emergence of shoulder features. These changes are consistent with partial reduction of Fe^3+^ and an increased contribution from Fe^2+^-enriched, FeO-like FeO_x_ local electronic environments (Fe^2+^-rich iron-oxide species) induced by oxygen loss. The intensity of the main peaks decreases, and new shoulder peaks emerge, suggesting that oxygen loss during the reduction process leads to changes in the local chemical environment of Fe. The enhancement of Fe^2+^ signals indicates that the chemical state of Fe becomes more complex in the reduced film, suggesting a more complex Fe local environment after reduction, potentially involving FeO_x_-like components.

The O K-edge spectra (Figure 5b) further support the reconstruction of Fe–O and Bi–O bonding. In the pristine film, the pre-edge feature at ~528.8 eV is attributed to O 2p–Fe 3d hybridization, while the higher-energy feature near ~533.3 eV is associated with O 2p states hybridized with Bi 6p-derived bands [42]. After reduction, the pre-edge intensity is markedly suppressed, consistent with weakened O 2p–Fe 3d hybridization and oxygen deficiency. In addition, a higher-energy feature becomes more pronounced (around ~537 eV), which may be related to surface oxygen species and secondary phases formed during the reduction process. Given the surface sensitivity of TEY and the coexistence of multiple oxygen species in chemically heterogeneous films, the O K-edge changes are also interpreted qualitatively rather than used to quantify an absolute oxygen deficiency. Overall, the Fe and O XAS results corroborate the XPS and diffraction observations, indicating substantial electronic-structure reconstruction driven by lattice-oxygen loss.

To study the changes in magnetic properties of BFO thin films before and after CaH_2_ reduction, M–H hysteresis loops were measured at 2 K, 50 K, 250 K, and 300 K by sweeping the magnetic field between ±2 T, and M–T curves were measured under an applied field of 0.25 T using a PPMS. As shown in Figure S6a, the pristine BFO thin film exhibits a discernible hysteresis loop at 2 K, indicating a ferromagnetic-like component in the magnetic response. Here, ‘ferromagnetic-like’ is used phenomenologically to describe the finite hysteresis remaining in the background-corrected M(H) curves (after subtracting the SrTiO_3_ substrate/sample-holder contributions and removing the high-field linear background), and it does not imply intrinsic long-range ferromagnetic ordering. Within the present measurement window (±2 T), the loop alone does not uniquely determine the microscopic origin of the hysteresis; possible contributions may include weak-ferromagnetic canting in epitaxial BiFeO_3_, defect-related magnetism, and minor FeO_x_-like components below the detection limit of laboratory thin-film XRD. With increasing temperature (50 K, 250 K, and 300 K), the hysteresis gradually narrows and the remanence decreases, suggesting progressive weakening of the ferromagnetic-like contribution [43].

For the reduced film (Figure S6b), a hysteresis loop is still discernible at 2 K; however, the loop amplitude, coercivity, and high-field magnetization are substantially suppressed compared with the pristine state. At 300 K, the loop becomes nearly closed, indicating that which is consistent with disruption/dilution of Fe–O–Fe exchange and increased non-hysteretic (largely linear) background contributions, rather than the emergence of a dominant ferromagnetic impurity phase. Although oxygen vacancies can enhance magnetic responses in some oxide systems, their effect in BFO is not universal and depends strongly on defect concentration/distribution and whether the perovskite Fe–O–Fe network remains intact. In the present CaH2 reduction window, the reduced films fall into a strong-reduction and chemical-reconstruction regime; oxygen removal is therefore expected to disrupt Fe–O–Fe connectivity, increase magnetic disorder, and introduce non-magnetic (or weakly magnetic) components/regions, leading to a net suppression of the ferromagnetic-like contribution rather than the emergence of a dominant ferromagnetic impurity phase. Consistent with the background-corrected analysis described above, the reduced film is largely dominated by a near-linear high-field background (which may include diamagnetic contributions from metallic Bi and other non-hysteretic phases), whereas the hysteretic component is strongly suppressed. The M-T curve of the BFO thin film sample is shown in Figure S6c. The magnetization is higher at low temperatures (<50 K) and gradually decreases with increasing temperature, showing a typical trend of ferromagnetism weakening with increasing temperature [44]. The rapid decrease in the curve suggests a temperature-weakened hysteretic component that is consistent with a weak-ferromagnetic contribution in epitaxial BFO (e.g., spin-canting-related net moment), while defect-related contributions (oxygen vacancies/local disorder) cannot be fully excluded based on the present measurements. Across the entire measurement range (2 K–300 K), the magnetic properties of the BFO thin film maintain a certain strength, reflecting the stability of its lattice oxygen environment and chemical state.

For the reduced BFO thin film sample, as shown in Figure S6d, the magnetization significantly decreases across the entire temperature range compared to the pre-reduction state, particularly in the low-temperature region (<50 K), indicating a substantial weakening of the ferromagnetic properties. The decrease in magnetization with increasing temperature becomes more gradual, suggesting that the magnetic order in the reduced thin film is disrupted by oxygen vacancies and changes in chemical states, leading to a reduction in ferromagnetic sources. In the high-temperature region (>200 K), the magnetization becomes nearly constant, suggesting that the reduced film is dominated by a much weaker magnetic response. Previous studies reported magnetization enhancement upon oxygen-vacancy engineering particularly when phase purity is preserved and/or interfacial reconstruction introduces additional exchange interactions or magnetic configurations. By contrast, CaH_2_-treated films fall into a stronger reduction/chemical-reconstruction regime, and the net effect is a suppression rather than an enhancement [45]. Overall, CaH_2_ treatment markedly suppresses the ferromagnetic-like component in BFO thin films, in line with the reduction-induced oxygen deficiency and reconstruction of the local chemical environment.

The UV–Vis absorption spectra of BFO thin films before and after CaH_2_ reduction show a pronounced modification of the optical response (Figure S7). Before reduction (Figure S7a), the film appears yellow and exhibits an absorption edge corresponding to a bandgap of ~1.7 eV, consistent with the semiconducting behavior of BFO, where optical absorption is dominated by intrinsic electronic transitions [46,47]. After CaH_2_ treatment at 365 °C for 2 h (Figure S7b), the film color changes from yellow to gray–black, accompanied by a red-shift in the absorption edge and enhanced sub-bandgap absorption. A Tauc analysis was performed assuming an indirect allowed transition for BFO (*n* = 2 in $\alpha h\nu ={A(h\nu -Eg)}^{n}$). The analysis yields an apparent optical gap of ~1.5 eV for the reduced film. Because the post reduction film is chemically and structurally heterogeneous, with possible contributions from oxygen-deficient regions, secondary phases, defect states, and enhanced scattering, the extracted value does not represent the intrinsic bandgap of a homogeneous BFO phase. It is therefore more appropriate to regard it as an apparent or effective optical onset used for qualitative comparison only. After CaH_2_ treatment, the film becomes chemically/structurally inhomogeneous and markedly roughened, such that oxygen-deficient regions, possible secondary phases, defect-/tail-state absorption, and enhanced scattering may contribute to the measured absorbance. Therefore, the linear fitting window in the Tauc plot is not necessarily unique, and the extracted value is model-dependent. In this context, the Tauc-derived value (~1.5 eV) is reported as an apparent/effective optical onset for qualitative comparison only, rather than an intrinsic bandgap of single-phase BFO.

## 4. Conclusions

This study shows that, under the present sealed-tube CaH_2_ reduction conditions, especially for the representative treatment at 365 °C for 2 h and within the investigated temperature range of 345 to 380 °C, epitaxial BFO thin films do not evolve into a stable homogeneous oxygen-deficient perovskite phase, but instead enter a chemically reconstructed multiphase state. Thin-film XRD provides the primary structural evidence, showing that the perovskite-related (00l) reflections of BFO deteriorate after reduction while new reflections attributable to Bi-containing secondary phases (e.g., Bi_2_O_3_/Bi^0^) emerge, consistent with decomposition and phase separation rather than a topotactic BiFeO_3-δ_ framework. These diffraction signatures are corroborated by the damping of XRR fringes and the AFM/STEM–EDS observations of increased surface roughness and pronounced near-surface chemical inhomogeneity. Consistently, XPS and XAS reveal reduction-induced chemical-state reconstruction, including the appearance of a lower-binding-energy Bi 4f doublet consistent with Bi^0^ formation and Fe spectral shifts indicative of an increased Fe^2+^ contribution under oxygen-loss conditions. As a consequence of this structural/chemical breakdown, the hysteretic magnetic component (after substrate/background correction) is strongly suppressed, consistent with disrupted Fe–O–Fe exchange connectivity and dilution by largely non-hysteretic secondary phases. The optical response is also markedly modified, showing a red-shifted absorption onset with enhanced sub-gap absorption; accordingly, any Tauc-derived “bandgap” for the reduced film is reported as an apparent/effective optical onset of a chemically heterogeneous film rather than an intrinsic bandgap of single-phase BFO.

These findings highlight a key limitation in using hydride-based reduction for perovskites containing easily reducible A-site cations like Bi^3+^. The differing reduction potentials of the cations promote decomposition over controlled topotactic oxygen removal. The present results carry important implications for the application-oriented development of BFO-based thin-film devices. Recent studies have demonstrated that precise engineering of defects, interfaces, and chemical states in oxide thin films can indeed unlock or enhance functional properties for dielectrics, spintronics, and optoelectronics [47]. In contrast, the present work delineates a stability boundary for one specific chemical approach—hydride-based topochemical reduction. This serves as a critical case study highlighting that not all defect-engineering routes are compatible with every material system. For device applications requiring long-term reliability, understanding and avoiding such chemical instability limits is as crucial as pursuing performance enhancement.

For future work, achieving a homogeneous reduced phase may require strategies to kinetically stabilize the A-site, such as designing more effective diffusion barriers or using alternative, milder reducing agents.

## Data Availability

The original contributions presented in this study are included in the article/Supplementary Material. Further inquiries can be directed to the corresponding authors.

## Supplementary Materials

The following supporting information can be downloaded at: https://www.mdpi.com/article/10.3390/ma19071310/s1, Figure S1: AFM analysis of a SrTiO_3_ (001) substrate after processing. (a) 1 μm × 1 μm AFM image; (b) Step height profile obtained from a line scan across (a); Figure S2: Control annealing without CaH_2_ under identical sealed-tube conditions; Figure S3: XRD patterns of BiFeO_3_ thin films reduced at different CaH_2_ treatment temperatures; Figure S4: First-principles results and formation-enthalpy analysis of BiFeO_3_. (a) Spin-polarized band structure of BiFeO_3_, where the black solid lines and red dashed lines denote the spin-up and spin-down states, respectively, indicating an indirect-band-gap semiconductor; (b) wave-vector dependence of the squared polarization magnitude (P2) along different k-point paths; (c) total density of states (DOS) and projected/partial density of states (PDOS); (d) schematic of the BiFeO_3_ crystal-structure model used in the calculations; Figure S5: AFM images of BiFeO_3_ thin films (a) before and (b) after reduction treatment; Figure S6: Magnetic properties of BiFeO_3_ thin films (a,c) before and (b,d) after reduction. (a,b) M-H curves measured at 2 K, 50 K, 250 K, and 300 K with the field swept between ±2 T. (c,d) M-T curves measured under a magnetic field strength of 0.25 T; Figure S7: The UV-Vis absorption spectra of BiFeO_3_ thin films (a) before and (b) after reduction with CaH_2_. (A photograph of the sample is located in the upper left corner).
