# Supplementary material for: Reduction Pathway and Temperature-Dependent Decomposition of Epitaxial BiFeO3 Thin Films Under CaH2 Treatment
Source: Materials (Basel). 2026 Mar 26;19(7):1310. doi: 10.3390/ma19071310 (PMC13073558; doi:10.3390/ma19071310)
Supplement: Supplementary file 1 [file materials-19-01310-s001.zip › materials-4141132-supplementary.pdf]

Supporting information for

# Reduction pathway and temperature-dependent decomposition of epitaxial $BiFeO_3$ thin films under $CaH_2$ treatment

**Jie Gong [1], Nian Li [1,*], Mahliya Lokman [2], Mengsha Li [2,*], Ke Zhang [1,*] and Liang Qiao [1]**

[1] School of Physics, University of Electronic Science and Technology of China, Chengdu 611731, China; jiegong@std.uestc.edu.cn (J.G.); liang.qiao@uestc.edu.cn (L.Q.)

[2] Center for Microscopy and Analysis, Nanjing University of Aeronautics and Astronautics,
Nanjing 210016, China; leah05@nuaa.edu.cn

* Correspondence: nianli@uestc.edu.cn (N.L.); limengsha@nuaa.edu.cn (M.L.); phyzk@uestc.edu.cn (K.Z.)

In this work, $SrTiO_3$ (001) substrates were etched using buffered hydrofluoric acid (BHF). The etching mechanism is based on the preferential reaction of SrO with BHF compared to $TiO_2$, forming water-soluble strontium hydroxide, $Sr(OH)_2$. After etching and nitrogen blow-drying, the substrates were annealed in a tube furnace to obtain a step-like surface morphology. Annealing was performed at 1000 °C for 1 hour to prevent further Sr diffusion from the bulk to the surface at higher temperatures. This combined wet etching and thermal annealing process induced surface atomic reconstruction, resulting in a clear step-and-terrace structure, as shown in the AFM image of Fig. S1a, indicating the formation of a $TiO_2$-terminated surface. The step height, determined from Fig. S1b, is 0.33 nm.

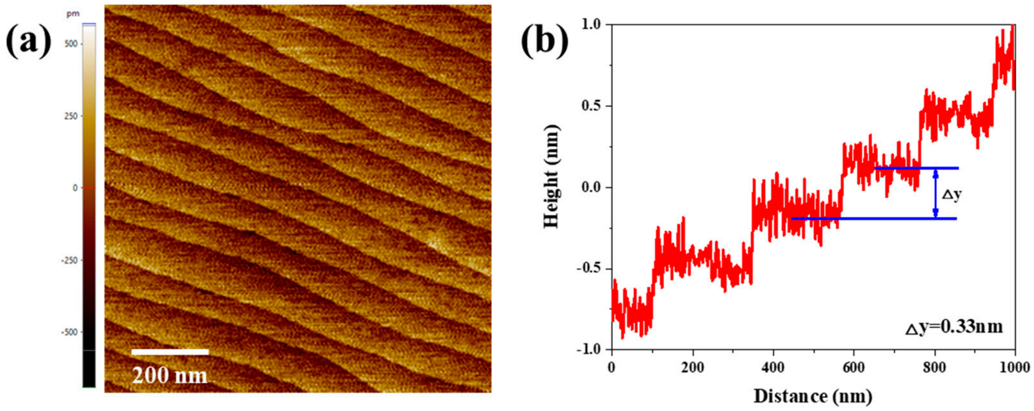


**Figure S1.** AFM analysis of a $SrTiO_3$ (001) substrate after processing. (a) 1 μm × 1 μm AFM image; (b) Step height profile obtained from a line scan across (a).

As a control, an identical sealed-tube anneal without $CaH_2$ shows no detectable secondary-phase reflections and no obvious peak-position changes (Fig. S2), indicating that the thermal history alone does not account for the decomposition signatures.

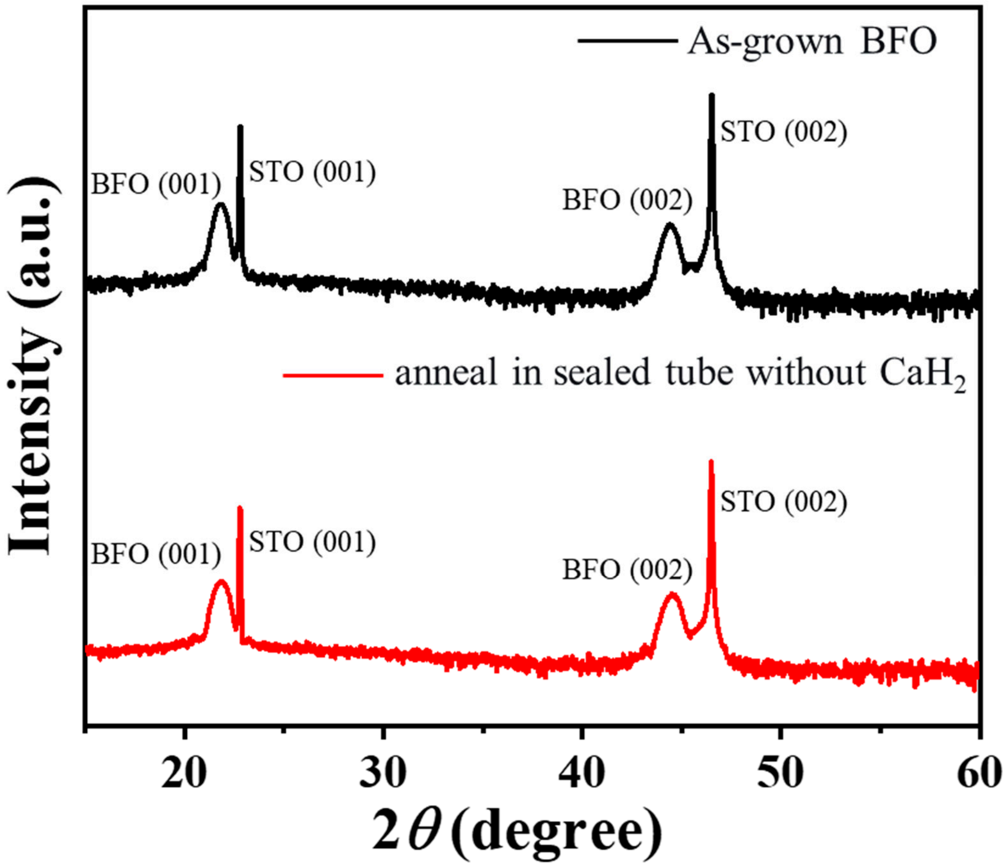


**Figure S2**. Control annealing without $CaH_2$ under identical sealed-tube conditions.

To further probe the kinetic role of reduction temperature, $CaH_2$ treatments were conducted for a fixed duration of 2 h over a temperature range of 345 to 380 °C and collected the corresponding XRD series (Fig. S3). With increasing temperature, the perovskite-related diffraction features progressively deteriorate while additional reflections emerge and strengthen, indicating a temperature-accelerated, threshold-like transition rather than a purely binary "before/after" change. This temperature series supports that, under the present sealed-tube $CaH_2$ configuration and time scale, decomposition and phase separation become dominant beyond a critical reduction severity.

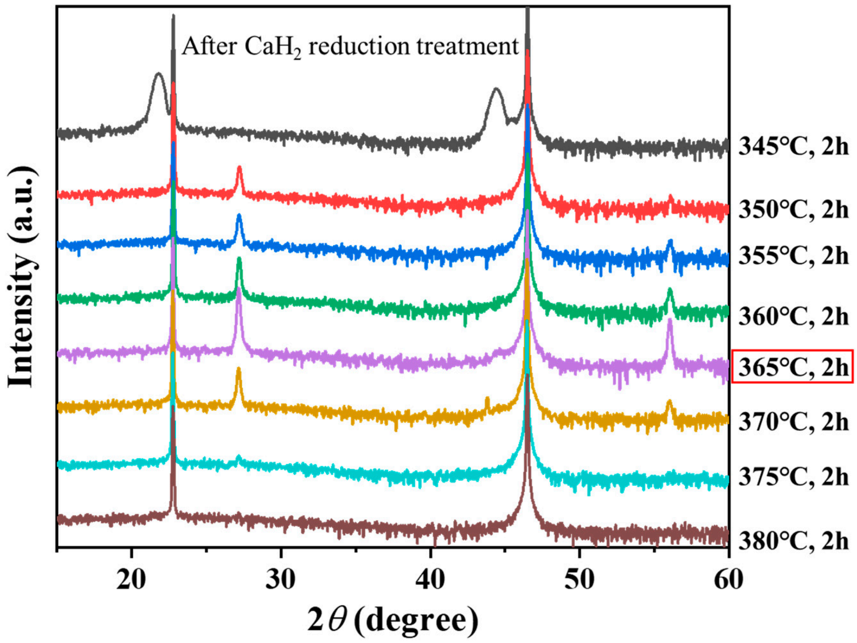


**Figure S3**. XRD patterns of $BiFeO_3$ thin films reduced at different $CaH_2$ treatment temperatures.

To rationalize the experimentally observed structural degradation from a thermodynamic perspective, first-principles calculations were performed for stoichiometric $BiFeO_3$ and an ideal deep oxygen-deficient limit, $BiFeO_2$, including analyses of the band structure, density of states, polarization response, and formation enthalpy (Fig. S4). Within computational framework, $BiFeO_3$ exhibits a clearly favorable (negative) formation enthalpy, whereas the deep oxygen-deficient $BiFeO_2$ limit shows a positive formation enthalpy, indicating that deep oxygen removal into a $BiFeO_2$-like perovskite is thermodynamically unfavorable and prone to destabilization. This energetic trend supports experimental observations that sufficiently strong $CaH_2$ reduction drives collapse and phase separation, highlighting the need to control temperature and time to avoid entering a deep oxygen-deficient regime. $BiFeO_2$ is used here as an idealized limiting model to evaluate stability trends, and the actual reduced state may evolve via multiphase pathways.

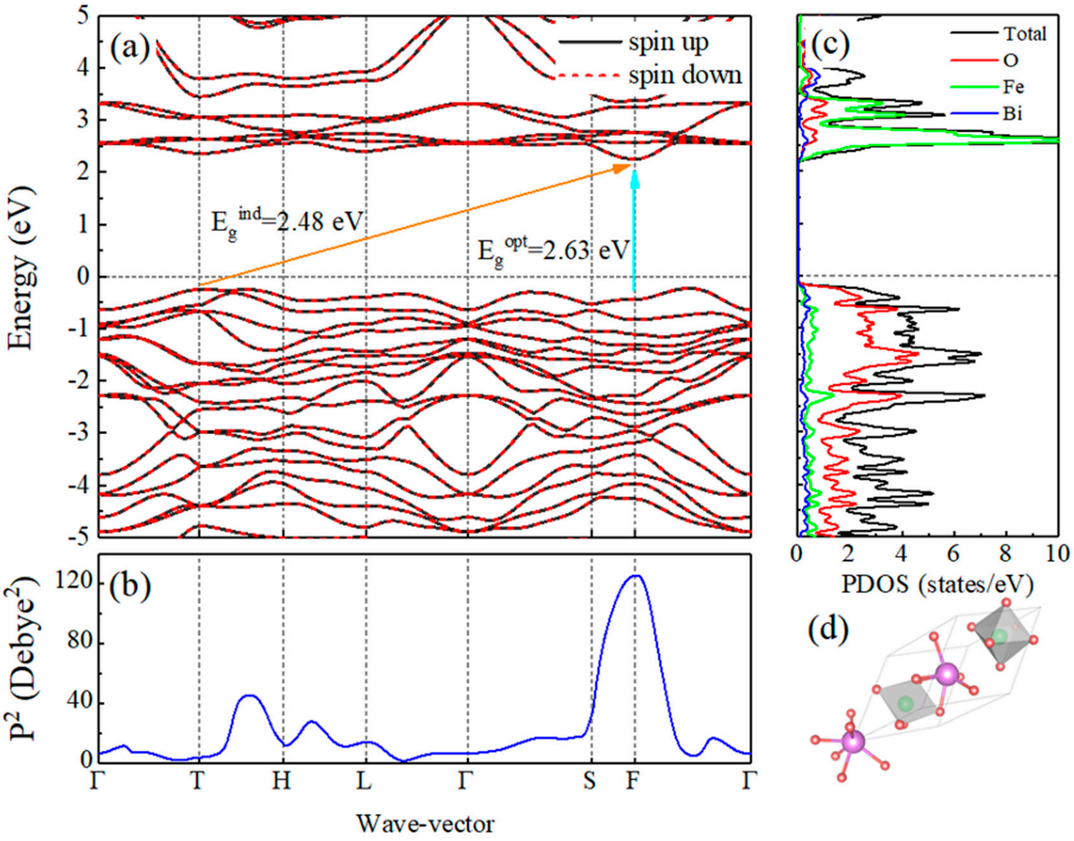


**Figure S4** First-principles results and formation-enthalpy analysis of $BiFeO_3$. (a) Spin-polarized band structure of $BiFeO_3$, where the black solid lines and red dashed lines denote the spin-up and spin-down states, respectively, indicating an indirect-band-gap semiconductor; (b) wave-vector dependence of the squared polarization magnitude ($P^2$) along different k-point paths; (c) total density of states (DOS) and projected/partial density of states (PDOS); (d) schematic of the $BiFeO_3$ crystal-structure model used in the calculations.

Atomic force microscopy (AFM) was used to examine the surface morphology evolution of BFO thin films before and after $CaH_2$ treatment (Fig. S5). As shown in Fig. S5a, the pristine film exhibits a granular surface morphology. The root-mean-square roughness (Rq) extracted from the AFM image is 1.817 nm, indicating nanoscale height variations on the film surface. After reduction, as shown in Fig. S5b, the surface morphology changes markedly from an irregular granular texture to a striped pattern. Concomitantly, Rq increases to 2.571 nm, suggesting an appreciable roughening of the surface. This morphological reconstruction is likely associated with reduction-driven changes in oxygen stoichiometry and local bonding, and may also be influenced by near-surface chemical/structural inhomogeneity and the possible precipitation of secondary-phase particles.

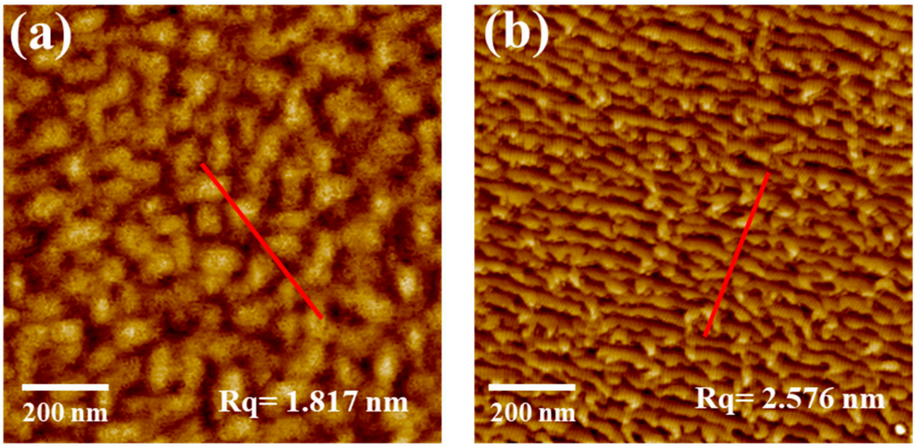


**Figure S5.** AFM images of $BiFeO_3$ thin films (a) before and (b) after reduction treatment.

To study the changes in magnetic properties of BFO thin films before and after $CaH_2$ reduction, M–H hysteresis loops were measured at 2 K, 50 K, 250 K, and 300 K by sweeping the magnetic field between ±2 T, and M–T curves were measured under an applied field of 0.25 T using a PPMS. As shown in Fig. S6a, the BFO thin film exhibits a clear hysteresis loop at 2 K with finite coercivity and remanent magnetization, indicating a ferromagnetic-like component in the magnetic response. With increasing temperature (50 K, 250 K, and 300 K), the hysteresis gradually narrows and the remanence decreases, suggesting progressive weakening of the ferromagnetic-like contribution.

For the reduced film (Fig. S6b), a hysteresis loop is still discernible at 2 K; however, the loop amplitude, coercivity, and high-field magnetization are substantially suppressed compared with the pristine state. At 300 K, the loop becomes nearly closed, indicating that the ferromagnetic-like contribution is strongly diminished after reduction. This suppression is consistent with reduction-driven oxygen loss and structural reconstruction evidenced by XRD/RSM and XPS/XAS, which can disrupt magnetic exchange pathways and introduce additional non-ferromagnetic contributions. The M-T curve of the BFO thin film sample is shown in Fig. S6c. The magnetization is higher at low temperatures (<50K) and gradually decreases with increasing temperature, showing a typical trend of ferromagnetism weakening with increasing temperature. The rapid decrease in the curve indicates that the magnetism of the BFO thin film mainly originates from highly ordered ferromagnetic phases, and its magnetism strongly depends on temperature. Across the entire measurement range (2K-300K), the magnetic properties of the BFO thin film maintain a certain strength, reflecting the stability of its lattice oxygen environment and chemical state.

For the reduced BFO thin film sample, as shown in Fig. S6d, the magnetization significantly decreases across the entire temperature range compared to the pre-reduction state, particularly in the low-temperature region (<50K), indicating a substantial weakening of the ferromagnetic properties. The decrease in magnetization with increasing temperature becomes more gradual, suggesting that the magnetic order

in the reduced thin film is disrupted by oxygen vacancies and changes in chemical states, leading to a reduction in ferromagnetic sources. In the high-temperature region (>200K), the magnetization becomes nearly constant, suggesting that the reduced film is dominated by a much weaker magnetic response. Overall, $CaH_2$ treatment markedly suppresses the ferromagnetic-like component in BFO thin films, in line with the reduction-induced oxygen deficiency and reconstruction of the local chemical environment.

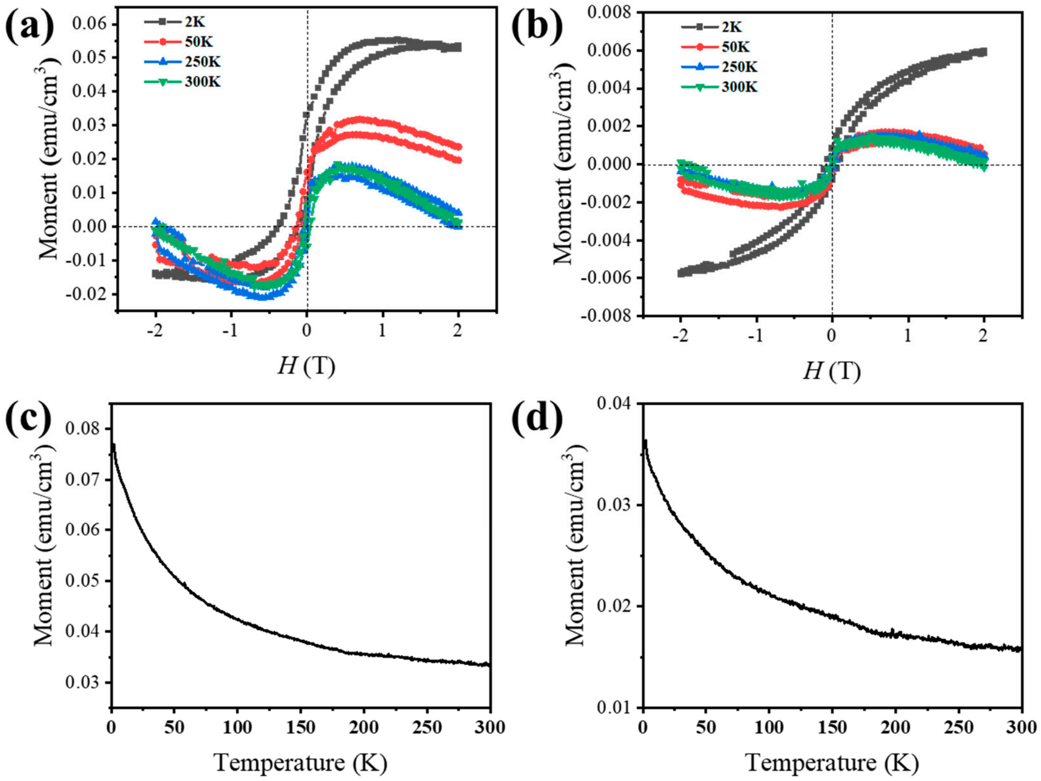


**Figure S6.** Magnetic properties of $BiFeO_3$ thin films (a, c) before and (b, d) after reduction. (a, b) M-H curves measured at 2 K, 50 K, 250 K, and 300 K with the field swept between ±2 T. (c, d) M-T curves measured under a magnetic field strength of 0.25 T.

The UV–Vis absorption spectra of BFO thin films before and after $CaH_2$ reduction show a pronounced modification of the optical response (Fig. S7). Before reduction (Fig. S7a), the film appears yellow and exhibits an absorption edge corresponding to a bandgap of ~1.7 eV, consistent with the semiconducting behavior of BFO, where optical absorption is dominated by intrinsic electronic transitions. After $CaH_2$ treatment at 365 °C for 2 h (Fig. S7b), the film color changes from yellow to gray–black, accompanied by a red-shift of the absorption edge and enhanced sub-bandgap absorption. A Tauc analysis was performed assuming an indirect allowed transition for BFO (n = 2 in $\alpha h\nu = A(h\nu - Eg)^n$). The analysis yields an apparent optical gap of ~1.5 eV for the reduced film. The Tauc approach assumes a relatively homogeneous absorber with a single dominant optical transition. After $CaH_2$ treatment, the film becomes chemically/structurally inhomogeneous and markedly roughened, such that

oxygen-deficient regions, possible secondary phases, defect-/tail-state absorption, and enhanced scattering may contribute to the measured absorbance. Therefore, the linear fitting window in the Tauc plot is not necessarily unique, and the extracted value is model-dependent. In this context, the Tauc-derived value (~1.5 eV) is reported as an apparent/effective optical onset for qualitative comparison only, rather than an intrinsic bandgap of single-phase BFO.

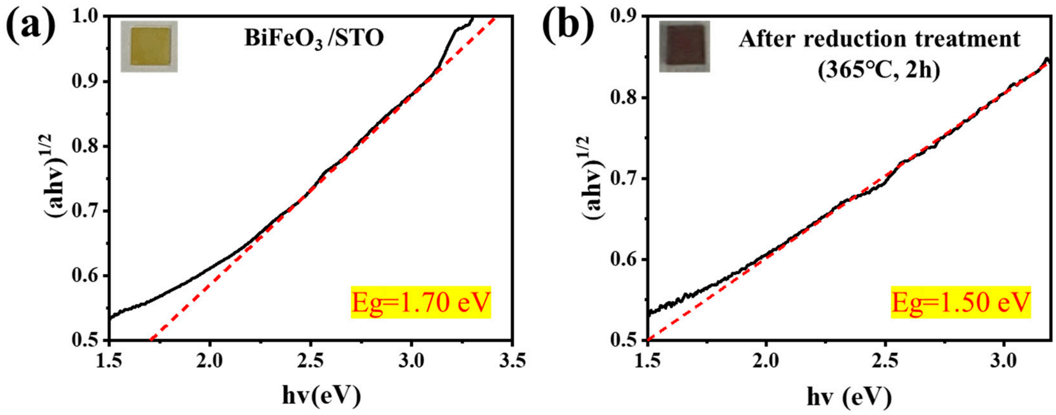


**Figure S7.** The UV-Vis absorption spectra of $BiFeO_3$ thin films (a) before and (b) after reduction with $CaH_2$. (A photograph of the sample is located in the upper left corner).
